# Supplementary figures and images for: Were climatic forcings the main driver for mid-holocene changes in settlement dynamics on the Varamin Plain (Central Iranian Plateau)?
Source: PLoS One. 2023 Oct 31;18(10):e0290181. doi: 10.1371/journal.pone.0290181 (PMC10617709; doi:10.1371/journal.pone.0290181)

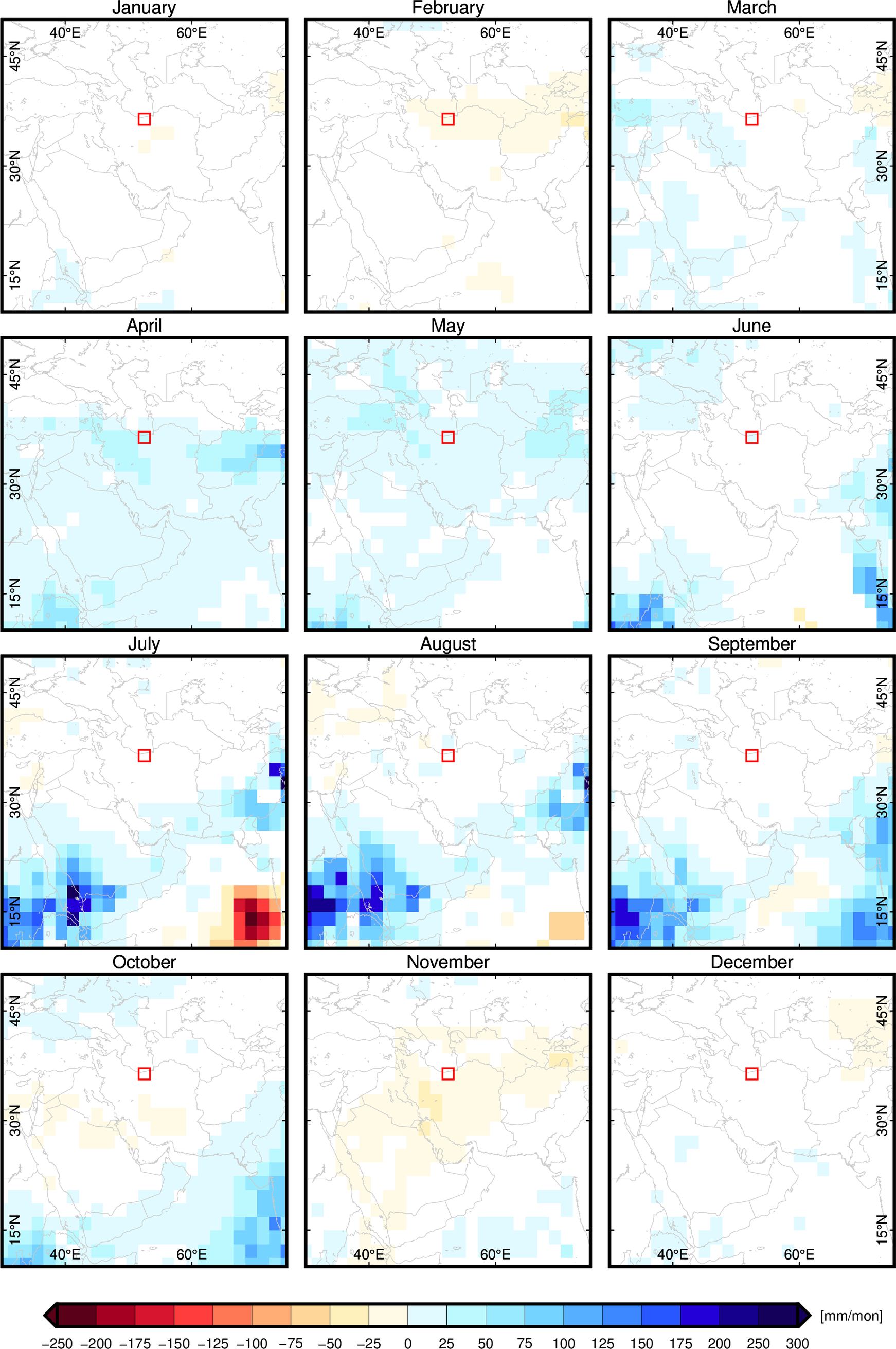

Supplement: S1 Fig — Please note, that all differences relate to the calendar in the model, i.e. modern calendar. (TIF) [file pone.0290181.s001.tif]

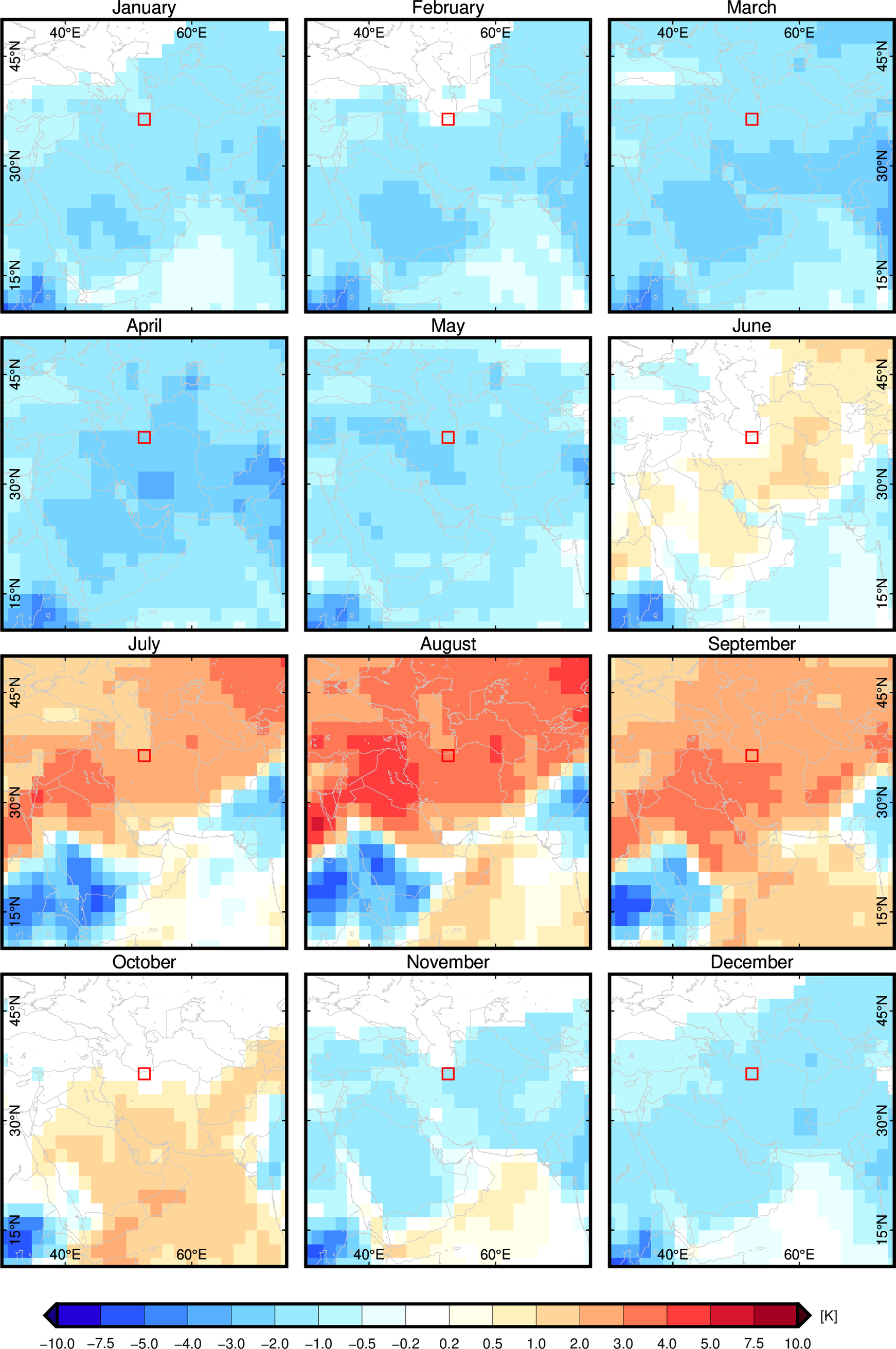

Supplement: S2 Fig — Please note, that all differences relate to the calendar in the model, i.e. modern calendar. (TIF) [file pone.0290181.s002.tif]

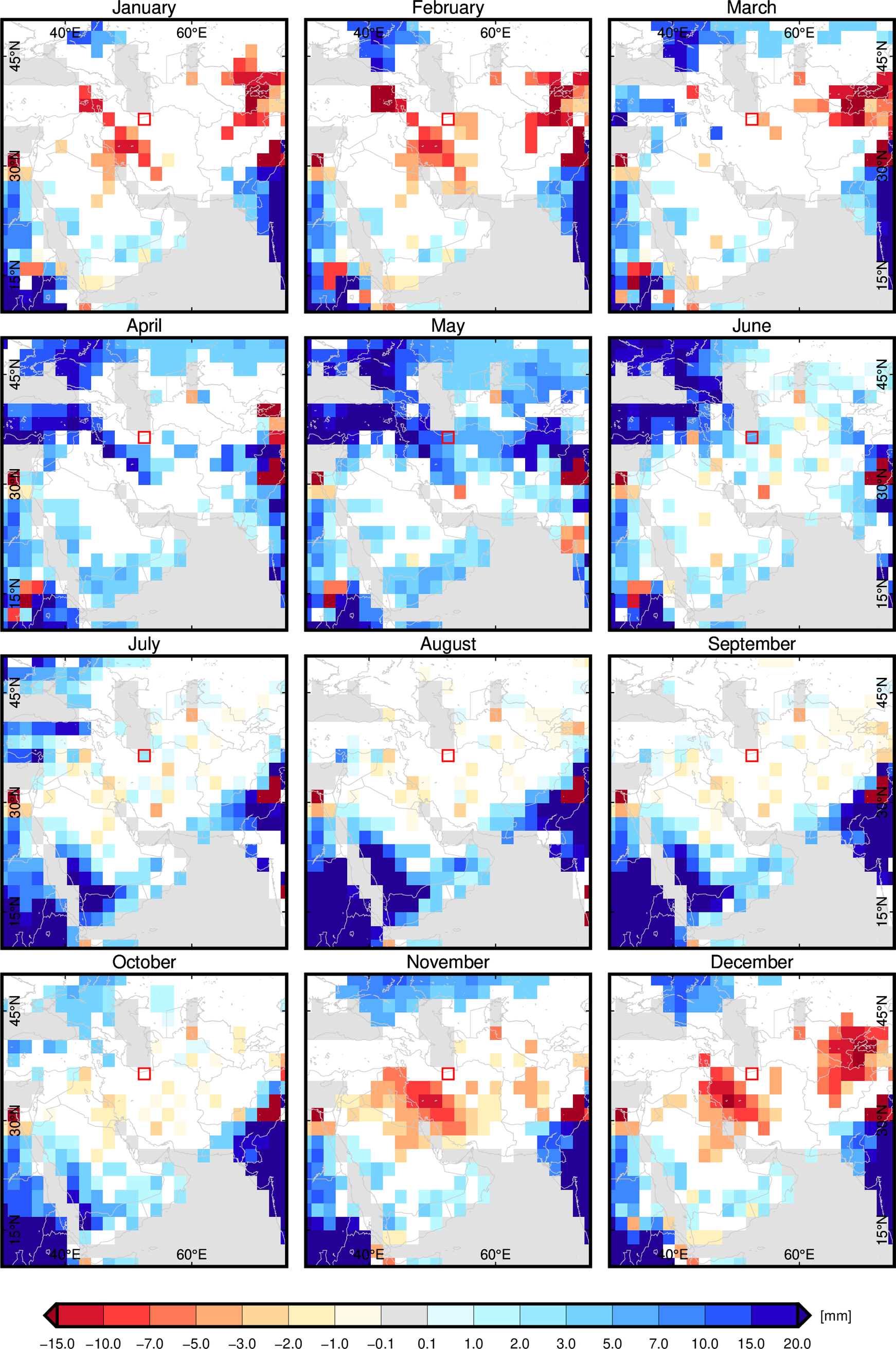

Supplement: S3 Fig — Please note, that all differences relate to the calendar in the model, i.e. modern calendar. (TIF) [file pone.0290181.s003.tif]
