# Supplementary material for: Were climatic forcings the main driver for mid-holocene changes in settlement dynamics on the Varamin Plain (Central Iranian Plateau)?
Source: PLoS One. 2023 Oct 31;18(10):e0290181. doi: 10.1371/journal.pone.0290181 (PMC10617709; doi:10.1371/journal.pone.0290181)
Supplement: Attachment — Submitted filename: KIRSTEN_Varamin plain climate settlement.docx [file pone.0290181.s005.docx]

In the article, “**Were climatic forcings the main driver for Mid-Holocene changes in settlement dynamics on the Varamin Plain (Central Iranian Plateau)?**”, Kirsten et al., present the results of a range of climate models and compare these with palaeoclimate records to examine past changes in precipitation, moisture transport, temperature and more. They then compare this evidence to existing data to explore impacts on water resources and settlement dynamics. I am not an expert in climate modelling, but overall this manuscript shows great promise with in-depth synthesis and analysis of data/findings from multiple disciplines. I really enjoyed reading the manuscript and think the conclusions are very important. I will be citing and using this article extensively once published! Having said that, the text itself has some significant issues (outlined below), mostly related to certain sections being too long whilst others needed further explanation. I would therefore recommend the manuscript for publication after major revisions and would like to re-review the manuscript once these are completed.

Major comments:

- Certain sections are far too long and detailed when compared to the contribution they make to the article. This makes the article overall too long in my opinion and the words could be better spent elsewhere. Section 2 (especially 2.1) is a key example of this, with lots of unnecessary information.
  Similarly, 4.1.2.1. has lots of explanation to reach the conclusion that there is no influence of the monsoon on the study region, which is to expected. If the section about moisture regime theories (4.1.2) was better laid out, it would be more clear and concise. I would suggest listing the theories you will cover in the first paragraph and then outlining them individually with a paragraph or two dedicated to each only.
- If there is space left in the wordcount, I would like to see a little more discussion/background on why settlement datasets are useful for comparison with climate data. And the best approaches for doing this. By connecting this to other similar studies, you could assess your own methods. For instance, recent research in other areas has looked at the impact of climate on settlement decline across very broad regions (e.g., Lawrence, Palmisano and Gruchy, 2021) and much smaller sub-regions (e.g., Fleitmann *et al.*, 2022; Jacobson *et al.*, 2022). The spatial extent of your project falls somewhere in between these studies, what are the pros and cons to this size of analysis?
  - These articles, and others globally (e.g., Groucutt *et al.*, 2022; Braun *et al.*, 2023), similarly find climate and other geographical factors as potentially contributing (but only weakly and as part of a large range of factors) to settlement growth and/or abandonment.
- Connected to this, there are other climate events linked to settlement/human changes that you could mention in the introduction again to look at pros/cons. For example, dry conditions from ~450 CE onwards are thought to have contributed to settlement decline in Yemen/Oman and SW Turkey (Fleitmann *et al.*, 2022; Jacobson *et al.*, 2022). The same drying probably impacted N Iran (evidenced by the Neor record) but societal impacts have not been properly explored (Sharifi *et al.*, 2015).
- In terms of palaeoclimate records, you should clearly outline your selection criteria for inclusion. A number of other records from the region come to mind that I wonder why were not included – E.g., other caves: Soreq, Hoti, Kuna Ba, LoNAP514, Uluu-Too. And non-speleothem records: the Jiroft peat sequence, Neor lake, Zeribar, Meribad, the Dead Sea etc.

The manuscript also requires another read through for English errors, I have listed a few below but these are only representative:

- Line 23, Abstract: “Settlement crises in [the] ancient cultures..”
- Line 60: “An (re-)increase…” is confusing, I would rephrase
- Line 70-72: unclear…
- Line 95 and 187: “aridisation” should be aridification
- Line 115: I would change “How did seasonality of the climate change?” to How did climate seasonality change? For clarity.
- Need to swap in δ for d18o
- Line 122: should read “correspond”
- Acronyms, such as Central Iranian Plateau (CIP), only need to be introduced once (in the very first instance. Here you are introducing some of them several times.
- Line 169: no close bracket
- Line 189: “sediments, and even soil formation[,] were identified”
- Line 195: space needed after km2
- Line 306: Theis
- Line 401: wet and dry period[s]
- Line 482: maybe change the second “seasonal” in this sentence to “annual” to avoid double word usage

Minor comments:

- Keywords are not appropriate as too long and specific, should be changed. Try things like: Earth System Model (ESM), pollen, settlement, climate change, Iran, archaeology etc.
- Are both precipitation and temp and effective moisture mentioned?
- Are all data available?
- List the total number of settlements in each period?
- Line 236: how precise is the chronology? It would be good to quantify this somehow
- Line 506-511: some references are required here to restate which records/articles suggest favourable/dry conditions etc.
- Line 625: you use the term correlation, but I don’t think this is a statistical observation? Maybe say “inverse pattern”
- Please check that all utilised records are referenced. I noticed that the Sofular (Fleitmann *et al.*, 2009), Jeita (Cheng *et al.*, 2015) and Mawmluh (Berkelhammer *et al.*, 2012) cave data you use in Figure 8 were missing references.

Figures:

- Overall, I like the figures but I think there are too many and some could be simplified.
- Some figures could be combined to save on the total number (e.g., 3 and 5, 8 and 11, OR 10 and 12)
- You also need to link to all the figures, supplementary figures and tables in-text (i.e., see Figure 1…).
- Captions require references to datasets used or to state that they appear in the text (which some of them do not, see above about Figure 8)

**Biblio**

Berkelhammer, M. *et al.* (2012) ‘An Abrupt Shift in the Indian Monsoon 4000 Years Ago’, in *Climates, Landscapes, and Civilizations*. American Geophysical Union (AGU), pp. 75–88. Available at: https://doi.org/10.1029/2012GM001207.

Braun, T. *et al.* (2023) ‘Decline in seasonal predictability potentially destabilized Classic Maya societies’, *Communications Earth & Environment*, 4(1), pp. 1–12. Available at: https://doi.org/10.1038/s43247-023-00717-5.

Cheng, H. *et al.* (2015) ‘The climate variability in northern Levant over the past 20,000 years’, *Geophysical Research Letters*, 42(20), pp. 8641–8650. Available at: https://doi.org/10.1002/2015GL065397.

Fleitmann, D. *et al.* (2009) ‘Timing and climatic impact of Greenland interstadials recorded in stalagmites from northern Turkey’, *Geophysical Research Letters*, 36(19), pp. 1–5. Available at: https://doi.org/10.1029/2009GL040050.

Fleitmann, D. *et al.* (2022) ‘Droughts and societal change: The environmental context for the emergence of Islam in late Antique Arabia’, *Science*, 376(6599), pp. 1317–1321. Available at: https://doi.org/10.1126/science.abg4044.

Groucutt, H.S. *et al.* (2022) ‘The 4.2 ka Event and the End of the Maltese “Temple Period”’, *Frontiers in Earth Science*, 9(January), pp. 1–23. Available at: https://doi.org/10.3389/feart.2021.771683.

Jacobson, M.J. *et al.* (2022) ‘Settlement, environment, and climate change in SW Anatolia: Dynamics of regional variation and the end of Antiquity’, *PLOS ONE*. Edited by A. Zerboni, 17(6), p. e0270295. Available at: https://doi.org/10.1371/journal.pone.0270295.

Lawrence, D., Palmisano, A. and Gruchy, M.W. de (2021) ‘Collapse and continuity: A multi-proxy reconstruction of settlement organization and population trajectories in the Northern Fertile Crescent during the 4.2kya Rapid Climate Change event’, *PLOS ONE*, 16(1), p. e0244871. Available at: https://doi.org/10.1371/journal.pone.0244871.

Sharifi, A. *et al.* (2015) ‘Abrupt climate variability since the last deglaciation based on a high-resolution, multi-proxy peat record from NW Iran: The hand that rocked the Cradle of Civilization?’, *Quaternary Science Reviews*, 123, pp. 215–230. Available at: https://doi.org/10.1016/j.quascirev.2015.07.006.
