# Supplementary material for: Were climatic forcings the main driver for mid-holocene changes in settlement dynamics on the Varamin Plain (Central Iranian Plateau)?
Source: PLoS One. 2023 Oct 31;18(10):e0290181. doi: 10.1371/journal.pone.0290181 (PMC10617709; doi:10.1371/journal.pone.0290181)
Supplement: Attachment — Submitted filename: KIRSTEN_Varamin plain climate settlement_Review2_comments.docx [file pone.0290181.s006.docx]

**Reviewer 2**

The ms from Kirsten et al. investigate the relationship between Holocene climatic changes and settlement patterns on the Central Iranian Plateau (CIP), and particularly on the Varanin Plain alluvial fan. The analyses is done by combining a transient comprehensive Earth System Model simulation (8 ka BP to pre-industrial, 200Km2 grid), a high-resolution (40 km2 grid) regional snapshot simulation and a synthesis of pollen-based climate reconstructions. Results are compared with proxy records from the wider Western-central Asia area (mostly speleothems). The ms examines the situation at 7 ka (when archaeological data show an increase in settlement density of the CIP/VP) compared to pre-industrial/modern time; and discusses both the general trend and the short-time changes highlighted by the simulation during the Middle-Late Holocene, in the context of the available paleoclimatic framework. Atmospheric patterns responsible for the simulated variability and the spatial heterogeneity of both simulation and proxy-based reconstruction are discussed.

The paper is of interest and the simulation results fit well with the available paleoclimatic context, suggesting that changes in the climate (mostly in the seasonality of the precipitation causing differences in water availability during spring-early summer) may have had an influence on settlement dynamics in the CIP/VP. Discrepancies between data and model are accurately reported and the model limitations (especially regarding the ability to capture the spatial heterogeneity of the climate due to the complex topography) are accounted for. The ms is well written and almost completely clear (also to non-modelling specialist), though a bit too long and with some repetitions; figures are of good quality. Overall, the ms can be published in Plos-One after moderate changes regarding two general points and several details (reported in the order as they appear in the ms)

Dear Reviewer,

Thank you very much for your encouraging and constructive review and we hope that you will be willing to re-review our (revised) manuscript. Based on your comments and those made by the other reviewer, we have revised the manuscript (see manuscript with track changes).

Please find direct replies to your comments on the manuscript in the document in blue colour, in each case right below your comment.

Best
Fabian, Anne & Co-Authors

General points:

1) PALEODATA-MODEL COMPARISON (and comparison with the Archaeological Dataset):

-I have some doubts on the use of the pollen-based LegacyClimate 2.0 dataset as a proper term of comparison/test of the model. I understand that it could be a nice synthesis to summarize global, general trends, but I’m not convinced that it can be used at this level of detail. Looking at the original publications it seems that the coverage of the study area (meaning CWA in its whole) is really poor. Moreover, pollen assemblages during the Middle-late Holocene are strongly influenced by anthropic activities, especially in CWA, making any reasoning about climatic influences on human societies a bit circular (i.e. is not possible to determine whether the pollen assemblages is modified by climate or by humans). Specifically, the harmonization to genus level made impossible to check the appearance of specific taxa regarded as strong indicators of anthropic modification to the local vegetation. This, in my opinion, made the discussion about the EOFs a bit speculative. I would consider removing it and rely instead on some recent syntheses (see e.g. Palmisano et al. 2021 Holocene regional population dynamics and climatic trends in the Near East: A first comparison using archaeo-demographic proxies. Quaternary Science Reviews, 252, 106739; and references therein). I understand the comparison will be more qualitative, but in my opinion also more reliable.

Thank you for raising this concern. We think that the inclusion of the pollen-based reconstructions from the LegacyClimate 2.0 dataset are a valuable addition to this study. We use the pollen-based reconstructions for validating the simulated climate in the broader regions. We do not infer any conclusions on the settlement dynamic from those records. The climate reconstructions were performed for each record individually and as such – although LegacyClimate being a global synthesis – the reconstructions can be also used on a (sub-)regional scale.

The fossil records compiled in LegacyClimate 2.0 were reported in multiple studies and derived from different natural archives. The harmonization approach guarantees that all records are handled consistently. Although losing taxonomic information when merging taxa together into a higher taxonomic level, it also increases the possibility of matching climate analogs in the modern and the fossil datasets. To ensure the quality of the reconstruction, we used the ratio between the constrained (λ_1_) and unconstrained (λ_2_) explained variance from the application of a canonical correlation analysis (CCA) to the modern training dataset (described in Herzschuh et al, 2023) in order to assess the explained variance in the modern pollen dataset by the used climate variables. The higher the λ_1_ vs. λ_2_-ratio in the spatial modern dataset, the higher is the chance that this target climate variable has also impacted vegetation over time and therefore contributed to the variation in the fossil pollen dataset. Overall, a ratio of >= 1 is considered to indicate reliable reconstructions (Juggins, 2013), which is the case for those records used in this study.

The data coverage in WCA is indeed poor, but we think that, nevertheless, these records can and should be used for model - data comparison, in particular keeping in mind that data coverage of other proxies are even worse and do not allow for quantitative reconstructions.

We do not expect an anthropogenic impact on the pollen-based climate reconstruction for the mid-Holocene. We checked the abundance of *Plantaginaceae* and *Rumex* which we see as indicator for grazing and, thus, human influence by intense animal husbandry (see attached figures). The abundances of these taxa are low for most sites during the mid-Holocene (<= 2% for *Rumex* and <= 6% for *Plantaginaceae*), which is the main period of our analysis. In the late Holocene, the pollen percentages of these taxa increase, particularly in the Eastern Mediterranean region. In the 3000 year time-slice, we found the highest abundances of Plantaginaceae in two records with 17.6% and 7.1%, respectively. We do not expect that these few records considerably affect the general trend in the PCs. Nevertheless, land use might be one of several reasons leading to differences in the pattern in model - data comparisons and the strength of the response seen in the reconstructions. We therefore included it as cause for local differences to the model.

*Juggins, S.: Quantitative reconstructions in palaeolimnology: new paradigm or sick science?, Quaternary Sci. Rev., 64, 20–32, https://doi.org/10.1016/j.quascirev.2012.12.014, 2013.*

-I do not find a proper comparison between the model output and the archaeological dataset. The general settlement dynamics are discussed in the intro, largely repeated in section 2.3 and recalled in a qualitative fashion in comparison with the model results. This is a fair approach, but it make section 3.1 a bit useless, as the archeological data are compared only qualitatively, and not quantitatively

The archaeological dataset was already discussed and published in Pollock et al. (2023). However, we re-aggregated the data to the time periods shown in Fig. 2 and also created a new figure. Unfortunately, a quantitative approach with a high(er) temporal resolution cannot be achieved based on the existing data set (as outlined in section 3.1). Furthermore, even with a higher temporal resolution of the archaeological dataset, a quantitative comparison to the climate model dataset would be problematic due to the nature of model and the limitation discussed in the respective section. We are well aware that assigning the archaeological data to the Materials-section of the manuscript may be a bit problematic, as we do not perform any further (quantitative) analysis with this dataset. However, we are convinced that it should not only be mention in the section “study area” as we use the chronology of the dataset as a point of (qualitative) comparison to the model data. As a consequence of all of this, section 3.1 is very short.

2) PAPER STRUCTURE AND LENGTH:

-The paper is too long and a bit repetitive. Several sections can be reduced and merged.
As example, Section 4.1.2 and its subsections can be easily reduced to few sentences, just saying that the model does support/does not support the different hypothesis. Similarly, the first part of section 4.2 is a repetition from the introduction, and also e.g. lines 791-797 are a repetition from the introduction and section 4.2. Also, the summary in the last section (5) is too long and detailed, you should consider that the reader arrives here straight after reading the text, so it sound like a long repetition. Other examples of repetition regards the archaeological sections (see above)

We agree that the repetition of information and the detailed discussions make the manuscript seem unnecessarily long, but we think that cuts (especially in the Discussion) would tend to reduce the quality of this multi-disciplinary paper. We have tried to write a paper that is interesting and relevant for the climate modelling community as well as the (geo)-archaeological community. Due to the multi-disciplinary nature of the study, we have decided not only to combine the results and discussion parts, but also to write a short introduction and a short synthesis of the results for each part of the discussion. The discussion requires to set the results in the scientific context, in which of course, some studies already mentioned in the introduction are taken up and discussed again. The repetitions and sometimes longer discussions are due to this structure, but we consider this structure necessary for the comprehensibility of the paper. We have therefore retained the detailed discussion, but have shortened individual text modules in the discussion (Sect 4.1.2.1 and 4.2) as well as the summary. With respect to repetitions on archaeological sections, please see response to the comment above.

Minor points:

line 178: please define Qanats in English

This is a well known term in the archaeology of Western Asia. For details, we included a reference.

line 263 please be consistent, use Ka BP and millennia BCE, I guess there is a typo here (2.8-2.2 ka BCE) done

line 306 “Theis” should be a typo done

line 347: I would say verying temporal resolution and related uncertainties done

Figures
Fig.1 the different areas quoted in lines 125-133 (VP-CIP-WCA) should be reported on fig. 1

The areas are actually quoted in the caption of fig 1 already. Please specify.

These areas do not have a clear outline, which is why we only sketch their outline in the text without marking them in the figure in order to avoid discussions about the correct outline.

Fig. 5 is quoted before of Fig. 4 (line 303). 🡪 mistake corrected

Alo, given that the two models have a different reference periods (PI for MPI-ESM and modern climate for ICON-NWP) I would show also the comparison between PI and Modern, to check whether differences are significant or not.
We are not sure that we understand this suggestion. The problem is that the MPI-ESM simulation ends at PI and the ICON-NWP simulation covers only 7ka. Thus we could only compare the PI climate simulated with MPI-ESM and the observational data. And these two curves are already displayed on the Figure.

Fig. 4 should be quoted in line 342 🡪 done

References:
I guess ref 10 and 11 are not the right ones here as they are quite old and both deals with long but relatively low resolution records from Soreq Cave speleothems. I would suggest to have a look to some recent compilations for the region (e.g. –see also general comments above)

Thank you for pointing this out. We have included some recent compilations for the region (see section 3.4). The limitations that you mention are exactly the ones that led us to the decision not to include these records/datasets in our analysis (as most other records in the region).

Ref 30 is only submitted, it cannot be quoted in the ref list until it is at least accepted

The manuscript is important for our study due to its archaeological and geomorphological content related to the study area (Varamin Plain). In the meantime, the manuscript has been revised and will be re-submitted soon. We think it will be also helpful for the reader to be able to directly access this study.
